# Supplementary material for: End Sequence Analysis Toolkit (ESAT) expands the extractable information from single-cell RNA-seq data
Source: Genome Res. 2016 Oct;26(10):1397–410. doi: 10.1101/gr.207902.116 (PMC5052061; doi:10.1101/gr.207902.116)
Supplement: Supplemental Material [file supp_gr.207902.116_Supp_Materials.tar.gz › ESAT-esat_v0.1/doc/html/interfaceumms_1_1core_1_1alignment_1_1_alignment.html]

ESAT: umms.core.alignment.Alignment Interface Reference


ESAT


- Main Page
- Related Pages
- Packages
- Classes
- Files

- Class List
- Class Index
- Class Hierarchy
- Class Members

All Classes Namespaces Files Functions Variables Enumerator Pages

- umms
- core
- alignment
- Alignment

Public Member Functions |
List of all members

umms.core.alignment.Alignment Interface Reference

Inheritance diagram for umms.core.alignment.Alignment:

[legend]

Collaboration diagram for umms.core.alignment.Alignment:

[legend]

|  |  |
| --- | --- |
| Public Member Functions | |
| String | getReadName () |
|  | |
| int | getFragmentStart () |
|  | |
| int | getFragmentEnd () |
|  | |
| Collection< Integer > | getFragmentSize (CoordinateSpace C) |
|  | |
| int | getMappingQuality () |
|  | |
| boolean | isPaired () |
|  | |
| boolean | isNegativeStrand () |
|  | |
| boolean | isChimera () |
|  | |
| boolean | isDuplicate () |
|  | |
| void | setDuplicateFlag (boolean duplicateFlag) |
|  | |
| Strand | getFragmentStrand () |
|  | |
| int | getLastFragmentPositionStranded () |
|  | |
| int | getFirstFragmentPositionStranded () |
|  | |
| int | getFragmentMidpoint (Annotation annot) |
|  | |
| Collection<?extends Window > | getFragment (CoordinateSpace C) |
|  | |
| Annotation | getReadAlignmentBlocks (CoordinateSpace C) |
|  | |
| int | getStart () |
|  | |
| int | getEnd () |
|  | |
| String | getChr () |
|  | |
| int | getAlignmentStart () |
|  | |
| int | getAlignmentEnd () |
|  | |
| boolean | isMapped () |
|  | |
| Object | getAttribute (String string) |
|  | |
| String | getReadSequence () |
|  | |
| double | getWeight () |
|  | |
| boolean | isProperPair () |
|  | |
| void | setProperPairFlag (boolean properPairFlag) |
|  | |
| SAMRecord | toSAMRecord () |
|  | |
| Collection< Annotation > | getReadAlignments (CoordinateSpace space) |
|  | |
| Collection< Alignment > | getReadMates () |
|  | |
| Collection<?extends Annotation > | getSpliceConnections () |
|  | |
| boolean | hasIndel () |
|  | |
| void | setFragmentStrand (TranscriptionRead strand) |
|  | |
| int[] | getIntervalBetweenReads () |
|  | |
| void | setHeader (SAMFileHeader header) |
|  | |
| SAMFileHeader | getHeader () |
|  | |
| Public Member Functions inherited from umms.core.annotation.Annotation | |
| int | getSAMStart () |
|  | |
| int | getSAMEnd () |
|  | |
| String | getReferenceName () |
|  | |
| String | getName () |
|  | |
| Strand | getOrientation () |
|  | |
| Strand | getStrand () |
|  | |
| boolean | hasOrientation () |
|  | |
| int | numBlocks () |
|  | |
| List<?extends Annotation > | getBlocks () |
|  | |
| List<?extends Annotation > | getBlocks (boolean oriented) |
|  | |
| int | length () |
|  | |
| int | getSize () |
|  | |
| int | size () |
|  | |
| int | getLengthOnReference () |
|  | |
| int | getOrientedStart () |
|  | |
| int | getOrientedEnd () |
|  | |
| double | getScore () |
|  | |
| boolean | isUnoriented () |
|  | |
| int | getReferenceCoordinateAtPosition (int positionInAnnotation) |
|  | |
| int | getPositionAtReferenceCoordinate (int referenceCoordinate) |
|  | |
| int | getReferenceCoordinateAtPosition (int positionInAnnotation, boolean ignoreOrientation) |
|  | |
| int | getPositionAtReferenceCoordinate (int referenceCoordinate, boolean ignoreOrientation) |
|  | |
| void | setStart (int start) |
|  | |
| void | setEnd (int end) |
|  | |
| void | setOrientation (char orientation) |
|  | |
| void | setOrientation (Strand orientation) |
|  | |
| void | setOrientedStart (int orientedStart) |
|  | |
| void | setOrientedEnd (int orientedEnd) |
|  | |
| void | setReferenceName (String refName) |
|  | |
| void | setName (String name) |
|  | |
| void | setScore (double score) |
|  | |
| boolean | equals (Annotation other) |
|  | |
| void | expand (int deltaStart, int deltaEnd) |
|  | |
| Annotation | trim (int deltaStart, int deltaEnd) |
|  | |
| void | shift (int delta) |
|  | |
| void | moveToCoordinate (int coordinateInReference) |
|  | |
| Annotation | copy () |
|  | |
| List< Annotation > | disect (Annotation a) |
|  | |
| List< Annotation > | disect (List<?extends Annotation > disectors) |
|  | |
| Annotation | minus (Annotation other) |
|  | |
| Annotation | minus (Collection<?extends Annotation > others) |
|  | |
| int | getDistanceTo (Annotation other) |
|  | |
| String | toUCSC () |
|  | |
| String | toBED () |
|  | |
| String | toBED (int r, int g, int b) |
|  | |
| String | toShortBED () |
|  | |
| String | toBEDGraph () |
|  | |
| String | getFullInfoString () |
|  | |
| boolean | overlaps (Annotation other, int buffer) |
|  | |
| boolean | overlaps (Collection<?extends Annotation > others, int buffer) |
|  | |
| boolean | overlaps (Annotation other) |
|  | |
| boolean | overlaps (Collection<?extends Annotation > others) |
|  | |
| boolean | overlaps (Annotation other, int buffer, boolean considerOrientation) |
|  | |
| boolean | overlaps (Annotation other, boolean considerOrientation) |
|  | |
| boolean | overlapsStranded (Annotation other) |
|  | |
| int | getOverlap (Annotation other) |
|  | |
| boolean | contains (Annotation other) |
|  | |
| Annotation | union (Annotation other) |
|  | |
| Annotation | intersect (Annotation other) |
|  | |
| List< Annotation > | intersect (List<?extends Annotation > annotations) |
|  | |
| int | compareToAnnotation (Annotation b) |
|  | |
| void | stitchTo (Annotation next) |
|  | |
| String | toCigar () |
|  | |
| boolean | fullyContains (Annotation annotation) |
|  | |
| Annotation | complement () |
|  | |
| boolean | equals (Annotation other, boolean useOrientation) |
|  | |
| int | getMidpoint () |
|  | |

## Detailed Description

Based on the Alignment interface in IGV. This interface represents any read or pair of reads aligned to the genome

Author
:   skadri

## Member Function Documentation

|  |  |  |  |  |
| --- | --- | --- | --- | --- |
| int umms.core.alignment.Alignment.getAlignmentEnd | ( |  | ) |  |

Returns the end position of the alignment

Returns

Implemented in umms.core.alignment.AbstractPairedEndAlignment, and umms.core.alignment.SingleEndAlignment.

|  |  |  |  |  |
| --- | --- | --- | --- | --- |
| int umms.core.alignment.Alignment.getAlignmentStart | ( |  | ) |  |

Returns the start position of the alignment

Returns

Implemented in umms.core.alignment.AbstractPairedEndAlignment, and umms.core.alignment.SingleEndAlignment.

|  |  |  |  |  |  |
| --- | --- | --- | --- | --- | --- |
| Object umms.core.alignment.Alignment.getAttribute | ( | String | *string* | ) |  |

Returns the value for the specified attribute

Parameters
:   |  |  |
    | --- | --- |
    | string |  |

Returns

Implemented in umms.core.alignment.SingleEndAlignment, and umms.core.alignment.AbstractPairedEndAlignment.

|  |  |  |  |  |
| --- | --- | --- | --- | --- |
| String umms.core.alignment.Alignment.getChr | ( |  | ) |  |

Get chromosome

Returns

Implements umms.core.annotation.Annotation.

Implemented in umms.core.alignment.AbstractPairedEndAlignment, and umms.core.alignment.SingleEndAlignment.

|  |  |  |  |  |
| --- | --- | --- | --- | --- |
| int umms.core.alignment.Alignment.getEnd | ( |  | ) |  |

Get end of the fragment

Returns

Implements umms.core.annotation.Annotation.

Implemented in umms.core.alignment.AbstractPairedEndAlignment.

Here is the caller graph for this function:

|  |  |  |  |  |
| --- | --- | --- | --- | --- |
| int umms.core.alignment.Alignment.getFirstFragmentPositionStranded | ( |  | ) |  |

Get the ending position of the read considering strand

Returns
:   Lowest position if negative strand, highest position otherwise

Implemented in umms.core.alignment.AbstractPairedEndAlignment, and umms.core.alignment.SingleEndAlignment.

|  |  |  |  |  |  |
| --- | --- | --- | --- | --- | --- |
| Collection<? extends Window> umms.core.alignment.Alignment.getFragment | ( | CoordinateSpace | *C* | ) |  |

This method returns the fragment formed by the alignment in the coordinate space C, specified.

Parameters
:   |  |  |
    | --- | --- |
    | C |  |

Returns

Implemented in umms.core.alignment.SingleEndAlignment, and umms.core.alignment.AbstractPairedEndAlignment.

|  |  |  |  |  |
| --- | --- | --- | --- | --- |
| int umms.core.alignment.Alignment.getFragmentEnd | ( |  | ) |  |

Returns the end position of the alignment in genomic space.

Returns

Implemented in umms.core.alignment.AbstractPairedEndAlignment, and umms.core.alignment.SingleEndAlignment.

|  |  |  |  |  |  |
| --- | --- | --- | --- | --- | --- |
| int umms.core.alignment.Alignment.getFragmentMidpoint | ( | Annotation | *annot* | ) |  |

Get midpoint of fragment with respect to coordinate space

Parameters
:   |  |  |
    | --- | --- |
    | annot | Parent annotation |

Returns
:   Fragment midpoint in coordinate space

Implemented in umms.core.alignment.SingleEndAlignment, and umms.core.alignment.AbstractPairedEndAlignment.

|  |  |  |  |  |  |
| --- | --- | --- | --- | --- | --- |
| Collection<Integer> umms.core.alignment.Alignment.getFragmentSize | ( | CoordinateSpace | *C* | ) |  |

Returns the fragment size (insert+lengths of the reads)

Returns

Implemented in umms.core.alignment.AbstractPairedEndAlignment, and umms.core.alignment.SingleEndAlignment.

|  |  |  |  |  |
| --- | --- | --- | --- | --- |
| int umms.core.alignment.Alignment.getFragmentStart | ( |  | ) |  |

Returns the start position of the alignment in genomic space.

Returns

Implemented in umms.core.alignment.AbstractPairedEndAlignment, and umms.core.alignment.SingleEndAlignment.

|  |  |  |  |  |
| --- | --- | --- | --- | --- |
| Strand umms.core.alignment.Alignment.getFragmentStrand | ( |  | ) |  |

Returns the strand for the fragment

Returns

Implemented in umms.core.alignment.SingleEndAlignment, and umms.core.alignment.AbstractPairedEndAlignment.

|  |  |  |  |  |
| --- | --- | --- | --- | --- |
| SAMFileHeader umms.core.alignment.Alignment.getHeader | ( |  | ) |  |

Returns the SAM file header for this alignment

Returns

Implemented in umms.core.alignment.AbstractPairedEndAlignment, and umms.core.alignment.SingleEndAlignment.

|  |  |  |  |  |
| --- | --- | --- | --- | --- |
| int [] umms.core.alignment.Alignment.getIntervalBetweenReads | ( |  | ) |  |

Get the coordinates of the first and last position of the interval between the reads

Returns
:   The coordinates of the space between the reads or null if single read or reads overlap

Implemented in umms.core.alignment.SingleEndAlignment, and umms.core.alignment.AbstractPairedEndAlignment.

|  |  |  |  |  |
| --- | --- | --- | --- | --- |
| int umms.core.alignment.Alignment.getLastFragmentPositionStranded | ( |  | ) |  |

Get the ending position of the read considering strand

Returns
:   Lowest position if negative strand, highest position otherwise

Implemented in umms.core.alignment.AbstractPairedEndAlignment, and umms.core.alignment.SingleEndAlignment.

|  |  |  |  |  |
| --- | --- | --- | --- | --- |
| int umms.core.alignment.Alignment.getMappingQuality | ( |  | ) |  |

Returns the mapping quality

Returns

Implemented in umms.core.alignment.SingleEndAlignment, and umms.core.alignment.AbstractPairedEndAlignment.

Here is the caller graph for this function:

|  |  |  |  |  |  |
| --- | --- | --- | --- | --- | --- |
| Annotation umms.core.alignment.Alignment.getReadAlignmentBlocks | ( | CoordinateSpace | *C* | ) |  |

This method returns the alignment blocks that the read actually aligns to.

Parameters
:   |  |  |
    | --- | --- |
    | C |  |

Returns

Implemented in umms.core.alignment.AbstractPairedEndAlignment, and umms.core.alignment.SingleEndAlignment.

|  |  |  |  |  |  |
| --- | --- | --- | --- | --- | --- |
| Collection<Annotation> umms.core.alignment.Alignment.getReadAlignments | ( | CoordinateSpace | *space* | ) |  |

This will return the actual read alignments

Parameters
:   |  |  |
    | --- | --- |
    | space | Coordinate Space to return the blocks in |

Implemented in umms.core.alignment.AbstractPairedEndAlignment, and umms.core.alignment.SingleEndAlignment.

|  |  |  |  |  |
| --- | --- | --- | --- | --- |
| Collection<Alignment> umms.core.alignment.Alignment.getReadMates | ( |  | ) |  |

This will return the actual read alignment objects NOT BLOCKS

Implemented in umms.core.alignment.SingleEndAlignment, and umms.core.alignment.AbstractPairedEndAlignment.

|  |  |  |  |  |
| --- | --- | --- | --- | --- |
| String umms.core.alignment.Alignment.getReadName | ( |  | ) |  |

This method returns the name of the alignment

Returns

Implemented in umms.core.alignment.SingleEndAlignment, and umms.core.alignment.AbstractPairedEndAlignment.

|  |  |  |  |  |
| --- | --- | --- | --- | --- |
| String umms.core.alignment.Alignment.getReadSequence | ( |  | ) |  |

Implemented in umms.core.alignment.AbstractPairedEndAlignment, and umms.core.alignment.SingleEndAlignment.

|  |  |  |  |  |
| --- | --- | --- | --- | --- |
| Collection<? extends Annotation> umms.core.alignment.Alignment.getSpliceConnections | ( |  | ) |  |

Return the splice connections contained within this read

Returns

Implements umms.core.annotation.Annotation.

Implemented in umms.core.alignment.SingleEndAlignment.

Here is the caller graph for this function:

|  |  |  |  |  |
| --- | --- | --- | --- | --- |
| int umms.core.alignment.Alignment.getStart | ( |  | ) |  |

Get start of the fragment

Returns

Implements umms.core.annotation.Annotation.

Implemented in umms.core.alignment.AbstractPairedEndAlignment.

Here is the caller graph for this function:

|  |  |  |  |  |
| --- | --- | --- | --- | --- |
| double umms.core.alignment.Alignment.getWeight | ( |  | ) |  |

The weight to give this alignment in the scoring. For example, if you want to scale by number of hits, the weight would be 1/NH

Returns

Implemented in umms.core.alignment.AbstractPairedEndAlignment, and umms.core.alignment.SingleEndAlignment.

Here is the caller graph for this function:

|  |  |  |  |  |
| --- | --- | --- | --- | --- |
| boolean umms.core.alignment.Alignment.hasIndel | ( |  | ) |  |

Returns whether the read contains an insertion or deletion

Returns

Implemented in umms.core.alignment.AbstractPairedEndAlignment, and umms.core.alignment.SingleEndAlignment.

|  |  |  |  |  |
| --- | --- | --- | --- | --- |
| boolean umms.core.alignment.Alignment.isChimera | ( |  | ) |  |

For single end, returns false. For paired end, returns true if reads successfully map to different chromosomes.

Returns

Implemented in umms.core.alignment.AbstractPairedEndAlignment, and umms.core.alignment.SingleEndAlignment.

|  |  |  |  |  |
| --- | --- | --- | --- | --- |
| boolean umms.core.alignment.Alignment.isDuplicate | ( |  | ) |  |

Returns true if the alignment is flagged as a duplicate

Returns

Implemented in umms.core.alignment.SingleEndAlignment, and umms.core.alignment.AbstractPairedEndAlignment.

|  |  |  |  |  |
| --- | --- | --- | --- | --- |
| boolean umms.core.alignment.Alignment.isMapped | ( |  | ) |  |

Returns true if the alignment is mapped

Returns

Implemented in umms.core.alignment.AbstractPairedEndAlignment, and umms.core.alignment.SingleEndAlignment.

|  |  |  |  |  |
| --- | --- | --- | --- | --- |
| boolean umms.core.alignment.Alignment.isNegativeStrand | ( |  | ) |  |

For paired end, returns true if the read in direction of transcription is negative stranded

Returns

Implements umms.core.annotation.Annotation.

Implemented in umms.core.alignment.AbstractPairedEndAlignment.

Here is the caller graph for this function:

|  |  |  |  |  |
| --- | --- | --- | --- | --- |
| boolean umms.core.alignment.Alignment.isPaired | ( |  | ) |  |

Returns true if the alignment is true.

Returns

Implemented in umms.core.alignment.SingleEndAlignment, and umms.core.alignment.AbstractPairedEndAlignment.

Here is the caller graph for this function:

|  |  |  |  |  |
| --- | --- | --- | --- | --- |
| boolean umms.core.alignment.Alignment.isProperPair | ( |  | ) |  |

Returns whether the alignment represented a proper pair defined by the aligner

Returns

Implemented in umms.core.alignment.AbstractPairedEndAlignment, and umms.core.alignment.SingleEndAlignment.

|  |  |  |  |  |  |
| --- | --- | --- | --- | --- | --- |
| void umms.core.alignment.Alignment.setDuplicateFlag | ( | boolean | *duplicateFlag* | ) |  |

Sets the isDuplicate Flag

Parameters
:   |  |  |
    | --- | --- |
    | duplicateFlag |  |

Implemented in umms.core.alignment.SingleEndAlignment, and umms.core.alignment.AbstractPairedEndAlignment.

|  |  |  |  |  |  |
| --- | --- | --- | --- | --- | --- |
| void umms.core.alignment.Alignment.setFragmentStrand | ( | TranscriptionRead | *strand* | ) |  |

Implemented in umms.core.alignment.SingleEndAlignment, and umms.core.alignment.AbstractPairedEndAlignment.

|  |  |  |  |  |  |
| --- | --- | --- | --- | --- | --- |
| void umms.core.alignment.Alignment.setHeader | ( | SAMFileHeader | *header* | ) |  |

Sets the SAM File header for this alignment

Parameters
:   |  |  |
    | --- | --- |
    | header |  |

Implemented in umms.core.alignment.AbstractPairedEndAlignment, and umms.core.alignment.SingleEndAlignment.

|  |  |  |  |  |  |
| --- | --- | --- | --- | --- | --- |
| void umms.core.alignment.Alignment.setProperPairFlag | ( | boolean | *properPairFlag* | ) |  |

Set whether the alignment represented a proper pair defined by the aligner

Returns

Implemented in umms.core.alignment.AbstractPairedEndAlignment, and umms.core.alignment.SingleEndAlignment.

|  |  |  |  |  |
| --- | --- | --- | --- | --- |
| SAMRecord umms.core.alignment.Alignment.toSAMRecord | ( |  | ) |  |

Implemented in umms.core.alignment.AbstractPairedEndAlignment, and umms.core.alignment.SingleEndAlignment.

Here is the caller graph for this function:

---

The documentation for this interface was generated from the following file:

- java/umms/core/alignment/Alignment.java


---

Generated on Fri Aug 15 2014 15:11:06 for ESAT by  

 1.8.7
